# Genome sequencing and analysis of isolates of *Cytospora sorbicola* and *Cytospora plurivora* associated with almond and peach canker

**DOI:** 10.1371/journal.pone.0334178

**Published:** 2025-10-17

**Authors:** Tawanda E. Maguvu, Rosa J. Frias, Florent P. Trouillas

**Affiliations:** 1 Department of Plant Pathology, University of California, Davis, California, United States of America; 2 Department of Plant Pathology, Kearney Agricultural Research and Extension Center, Parlier, California, United States of America; Friedrich Schiller University, GERMANY

## Abstract

*Cytospora sorbicola* and *C. plurivora* are significant canker pathogens that affect stone fruits and several other woody plants, severely limiting orchard productivity and longevity. These pathogens are challenging to control, and once established, there is no cure. Therefore, breeding for resistance is the most promising strategy to mitigate their impact. The first step in this process is understanding the interactions between the plants and the pathogens. To facilitate this understanding, we present genome sequencing and analysis of two isolates of *C. sorbicola* and two isolates of *C. plurivora*. The genomes of the *C. plurivora* isolates were assembled to a mean size of 38.4 ± 0.275 Mbp, with a mean guanine-cytosine (GC) content of 51.02 ± 0.25%. In contrast, the genomes of *C. sorbicola* were assembled to a mean size of 40.48 ± 0.11 Mbp, with a GC content of 50.28 ± 0.1%. The predicted number of protein coding genes for *C. plurivora* and *C. sorbicola* isolates was 9,878 ± 17.5 and 10,161 ± 34.5, respectively. Our genome mining efforts focused on identifying putative virulence determinants. We describe the distribution of identified secreted carbohydrate-active enzymes (CAZymes), transporters, cytochrome P450 monooxygenases, proteases, and genes encoding proteins involved in secondary metabolite synthesis. Additionally, we predicted potential effector proteins associated with these isolates. Gene Ontology analysis revealed a higher prevalence of transporter and peptidase activities, indicating an evolutionary adaptation to low pH and nutrient-limited environments. Although the primary aim of this study was to present the genome sequences, a phylogenomic analysis based on conserved single-copy gene sequences strongly suggests that *C. paraplurivora* is a species of *C. plurivora*. Therefore, we recommend amending the classification of *C. paraplurivora* sp. nov. (Ilyukhin et al. 2023) to *C. plurivora*. Most importantly, we present high-quality genome assemblies for *C. sorbicola* and *C. plurivora* isolates, which are critical resources for future studies on plant-pathogen interactions.

## Introduction

*Cytospora* species are destructive fungal pathogens (Ascomycota, Sordariomycetes, Diaporthales, Cytosporaceae) that cause cankers and dieback in woody plants across natural and agricultural ecosystems [1–5]. These pathogens lead to chronic wood infections in stone fruits, pome fruits, and nut crops [2,4,5], significantly limiting orchard productivity and longevity. The primary infection sites for most canker pathogens, including Cytospora, are pruning wounds [2,6–8]. Pruning is a crucial cultural practice in orchard production systems, as it helps maintain tree structure and improve fruit yield, making it an unavoidable process [9]. Several treatments are available to protect pruning wounds and reduce the risk of infection [10]. However, most effective chemical controls have undesirable environmental impacts. Therefore, breeding resistant plant varieties is considered the most effective and environmentally friendly approach to mitigate the effects of pathogens like Cytospora. To successfully breed resistant plants, it is essential first to understand the interactions between plants and pathogens [11–14]. Currently, there are no publicly available genomes for *Cytospora* species such as *C. sorbicola* and *C. plurivora*, which limits research in this area and the development of resistant plant cultivars.

Virulence factors in canker pathosystems include carbohydrate-active enzymes (CAZymes), membrane transporters, cytochrome monooxygenases, effector proteins, and secondary metabolites along with their associated toxins [4,15–19]. CAZymes play an essential role in the synthesis, modification, and degradation of polysaccharides, which is crucial for breaking down the host cell wall during infection [15,16]. Cytochrome monooxygenases help fungi adapt to diverse ecological niches by mitigating harmful chemical and biological signals [20]. Effectors assist in evading host defenses by concealing the pathogen’s presence or directly damaging host cells [21]. Transporters are vital for antifungal resistance and nutrient transport, both of which are essential for disease development in response to reactive oxygen species [22,23]. These virulence determinants can be identified through genomic sequencing, providing valuable insights into future studies on plant-pathogen interactions. Recent advances in whole genome sequencing, along with various bioinformatics tools, offer unprecedented opportunities for generating and analyzing genomic data on plant pathogens. Our laboratory has recently utilized these advancements to create well-annotated genomes for the phytopathogens *Ceratocystis destructans* and *Colletotrichum karsti* [24,25].

Additionally, genome sequencing and genetic data can reveal the signatures of speciation, which is particularly important for the genus *Cytospora*, where a lack of sequence data has hindered robust phylogenetic reconstructions [2,26,27]. Several studies have applied the phylogenomic species concept based on multi-locus sequence typing, leading to improved taxonomic clarification of cryptic fungal species [26,27]. However, most of these studies utilized a maximum of four loci, which have shown a significant probability of supporting conflicting topologies [28–30]. In contrast, analyses of entire datasets of concatenated genes from whole genome sequences produce a single, fully resolved species tree with maximum support and comparable results can be obtained by concatenating a minimum of 20 genes [29]. Limited data availability is a primary cause of phylogenetic inconsistencies. Due to this limitation, the taxonomy of the genus *Cytospora* has primarily relied on the morphological species concept [2]. While this concept has advanced the taxonomy of *Cytospora*, its limitations are well-documented [1,2]. For example, morphological overlaps among species are common, as is variability in morphology [1].

In this study, we sequenced and analyzed the genomic structure of two strains of *C. sorbicola* and two strains of *C. plurivora*, isolated from almond (*Prunus dulcis*) and peach (*Prunus persica*), respectively. Currently, there are no publicly available genomes for *C. sorbicola* and *C. plurivora*, hindering studies such as plant pathogen interaction, comparative genomics, and whole genome sequence-based phylogenomic. These studies have important implications in both basic and applied research. Additionally, our phylogenomic analysis based on conserved single-copy gene sequences strongly suggests that *C. paraplurivora* sp. nov (Ilyukhin et al. 2023) [31] is a species of *C. plurivora*. Therefore, we recommend amending the classification of *C. paraplurivora* sp. nov. to *C. plurivora*.

## Materials and methods

### Biological materials, genomic DNA extraction, and sequencing

*Cytospora sorbicola* isolates, identified as KARE228 and KARE59, were obtained from almond (*Prunus dulcis*) trees exhibiting symptoms of canker and dieback. Additionally, *C. plurivora* isolates KARE79 and KARE80 were isolated from peach trees (*Prunus persica*) showing similar symptoms. All isolates were collected in California. For detailed descriptions of the symptoms, geographical origins, isolation procedures, and other related metadata, please refer to Lawrence et al. 2018 [2]. Genomic DNA was extracted from the mycelium, which was scraped with a sterile scalpel from the surface of 14-day-old colonies, and hyphal tip-purified single isolates. This was performed using the FastDNA Spin Kit (MP Biomedicals, Irvine, CA), following the manufacturer’s protocol. The integrity and purity of the resulting DNA were assessed using agarose gel electrophoresis and NanoDrop spectrophotometry (ND-100; NanoDrop Technologies Inc., Wilmington, DE, USA), respectively. The DNA was then submitted for whole-genome sequencing to the Microbial Genome Sequencing Center (MIGS) in Pittsburgh, PA. Paired-end reads (2 × 151 bp) were generated using Illumina sequencing technology. Briefly, Illumina sequencing libraries were prepared using a tagmentation-based method along with a PCR-based Illumina DNA Prep kit and custom IDT 10 bp unique dual indices, targeting an insert size of 280 bp. No additional DNA fragmentation or size selection steps were performed. The sequencing was conducted on an Illumina NovaSeq X Plus sequencer.

### Assembly, and gene prediction

Unless otherwise noted, default parameters were used for all software. We obtained high-quality filtered reads with a quality score of Q > 20. The assembly of the quality-filtered reads was conducted using SPAdes version 3.15.3 [32]. To evaluate the quality of the assembled genomes, we utilized QUAST version 4.4 [33]. The completeness of the assembled genomes was assessed using the Benchmarking Universal Single-Copy Orthologs (BUSCO v5.2.2) software, benchmarking against fungi_odb10, and ascomycota_odb10 [34]. Prior to gene prediction, we performed repeat masking with RepeatMasker version 4.1.1. Gene prediction was executed using a combination of Augustus and GeneMark-ES, following the GenSAS eukaryotic annotation pipeline [35] as previously described [24,25], with no modifications. For generating consensus gene predictions, we used EvidenceModeler (EVM) to compile a consensus gene set from the predictions made by Augustus and GeneMark-ES [36].

### Annotations

To identify potential effector proteins from the proteome, SignalP v6.0 [37], TargetP v2.0 [38], and Deep THMM v2.0 [39] were employed to predict secretory proteins. From the pool of secreted proteins, EffectorP v3.0 was utilized to identify putative effector proteins [40]. Carbohydrate-active enzymes (CAZymes) were predicted using dbCAN3 v3.0.2 [41]. Fungal antiSMASH v7.0 was used to predict genes encoding proteins involved in secondary metabolite synthesis [42]. To annotate cytochrome P450 proteins from the proteome, the Fungal Cytochrome P450 Database (FCPD) was utilized [43], using the Phylum Ascomycota P450 sequences database with a BLASTP e-value of ≤ 10 and ≥ 44% amino acid identity. The Transporter Classification Database was used for predicting transporters [44]. Peptidases were identified using the MEROPS database [45]. Proteins were further annotated using BLAST+ against the NCBI non-redundant fungi protein database.

### Gene content comparisons and phylogenomic analyses

To compare the predicted proteins across isolates, the Orthovenn3 server was employed for whole-genome comparison and annotation of orthologous clusters [46]. The Orthovenn3 output also includes Gene Ontology (GO) annotations. The GO (Gene Ontology Resource) is a comprehensive source of functional information on gene products that leverage domain-specific ontologies [47]. By default, OrthoVenn3 conducts development and evolution analysis based on single-copy genes. OrthoVenn3 utilizes FastTree2 [48] to construct a phylogenetic evolutionary tree by maximum likelihood method [49]. In addition, the genome-based distance matrix calculator (http://enve-omics.ce.gatech.edu/g-matrix/index) was used to calculate average amino acid identity. For synteny analysis, D-GENIES [50] was used to visualize the dot plots using Minimap2 [51] with the default settings, the final plots were filtered for strong precision. The genomes of *Cytospora piceae* (GCA_016508685.1), *C. mali* (GCA_023079475.1), *C. paraplurivora* (GCA_021272945.2), and *C. leucostoma* (GCA_003795295.1) were included for phylogenomic analysis.

For further phylogenomic analysis, multi-locus sequence analysis was performed using the housekeeping genes ITS, *act*, and *tef1*. Sixteen isolates representing *C. plurivora* subclades as described by Lawrence et al. (2018), along with six closely related species, were included. Gene sequences were downloaded from NCBI, with accession numbers listed in [2]. All sequences were aligned using MAFFT [52], edited, and concatenated in MEGA-12 [53]. For Maximum Parsimony analysis, evolutionary history was inferred using the Subtree-Pruning-Regrafting (SPR) algorithm at search level 1, with initial trees generated by random addition of sequences (10 replicates) [54]. The analysis included 27 nucleotide sequences, applying the partial deletion option to remove all positions with less than 95% site coverage. For Maximum Likelihood analysis, the phylogeny was inferred using the Tamura-Nei model [55]. The initial tree for heuristic search was selected based on the higher log-likelihood between a Neighbor-Joining (NJ) and a Maximum Parsimony (MP) tree. The NJ tree was generated from a matrix of pairwise distances computed using the Tamura-Nei model [55], while the MP tree was the shortest among ten searches, each starting from a randomly generated tree. All evolutionary analyses were conducted in MEGA-12 [53]. All analyses were performed with 1,000 bootstrap replicates.

## Results and discussion

### Genome sequencing and assembly

The genomes of the *Cytospora plurivora* isolates KARE79 and KARE80 were assembled to a mean genome size of 38.4 ± 0.275 Mbp, with a mean guanine-cytosine (GC) content of 51.02 ± 0.25%. The quality metrics of the assembled *C. plurivora* genomes are provided in Table 1. In comparison, the genomes of the *C. sorbicola* isolates KARE59 and KARE228 were assembled to a mean genome size of 40.48 ± 0.11 Mbp, with a GC content of 50.28 ± 0.1%. The quality metrics for the assembled *C. sorbicola* genomes are also shown in Table 1. The genome sizes of both the *C. plurivora* and *C. sorbicola* isolates fall within the range of other publicly available *Cytospora* genomes. An assessment of genome integrity using BUSCO indicated that most conserved core fungi and core Ascomycota genes were predicted to be present in our genomes, suggesting that the genomes presented in this study are nearly complete (Table 1). Specifically, the isolates of *C. plurivora* contained at least 97.6% of the core Ascomycota genes, while the *C. sorbicola* isolates contained at least 96.3% (Table 1).

**Table 1:** Quality metrics of the assembled *Cytospora sorbicola* and *C. plurivora* genomes.

	*C. sorbicola* (KARE228)	*C. sorbicola* (KARE59)	*C. plurivora* (KARE79)	*C. plurivora* (KARE80)
Assembly Size (Mb)	40.59	40.37	38.16	38.71
Scaffolds	399	477	367	358
Scaffold N50 (Kb)	261,919	224,606	348,783	329,432
Scaffold L50 Count	48	52	34	35
GC (%)	50.22	50.33	51.27	50.77
Total Protein Coding Genes	10,126	10,195	9,860	9,895
BUSCO Estimated Completeness (%)	95.2 (96.8)	94.7 (96.3)	96.5(98)	96.1 (97.6)
Total Predicted Secretome	798	812	817	835
Predicted Putative Effectors	209	208	197	214

BUSCO values in parenthesis are benchmarking with *Ascomycota* whereas, the other value benchmarks with fungi in general

### Gene content and Gene Ontology (GO) distributions

**Fig 1:**
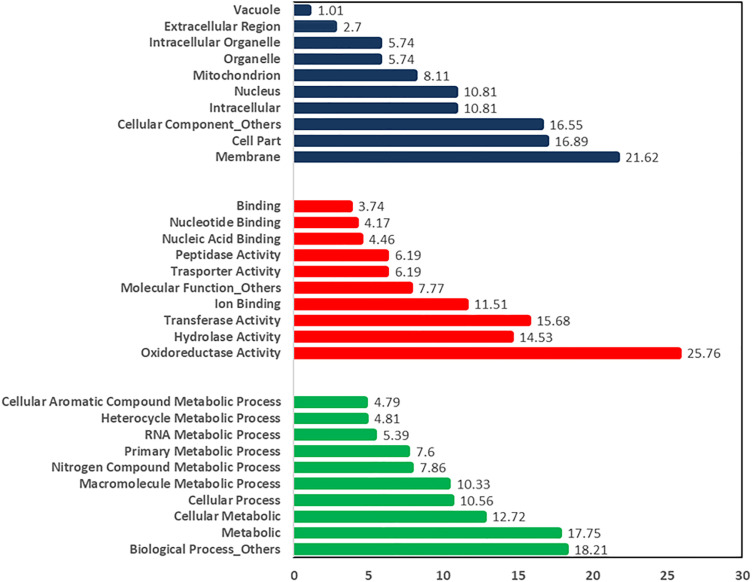
Relative abundance of the characterized functional categories of the clusters of orthologous genes (COG) for *C. plurivora* KARE79. The cellular component category is highlighted in blue, the molecular function category is highlighted in red, and the biological process category is highlighted in green.

**Fig 2:**
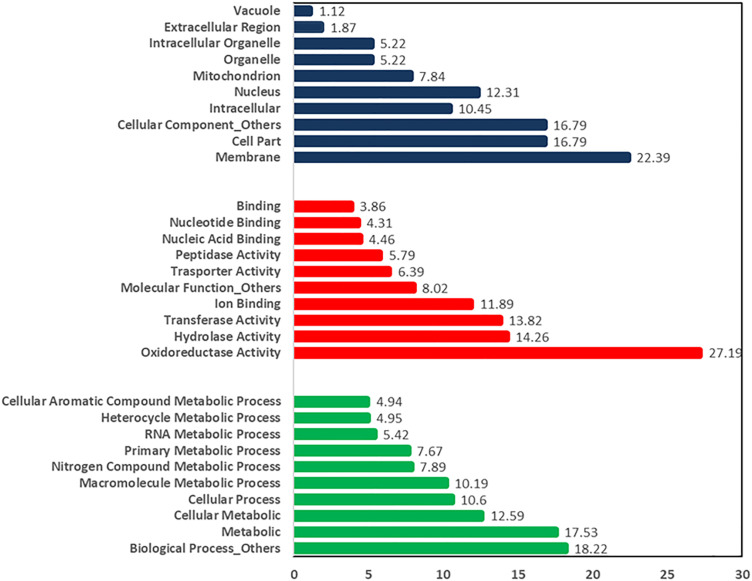
Relative abundance of the characterized functional categories of the clusters of orthologous genes (COG) for *C. sorbicola* KARE228. The cellular component category is highlighted in blue, the molecular function category is highlighted in red, and the biological process category is highlighted in green.

In this study, the predicted number of protein-coding genes was 9,860 for the *C. plurivora* isolate KARE79 and 9,895 for isolate KARE80 (Table 1). In comparison, the *C. sorbicola* isolates KARE59 and KARE228 had 10,195 and 10,126 predicted protein-coding genes, respectively (Table 1). The Gene Ontology (GO) resource provides a comprehensive source of functional information on gene products by utilizing domain-specific ontologies [47]. The GO annotations indicate that for isolate KARE79, the distribution of dominant annotations in the biological process category was as follows: 17.75% for metabolic processes, 12.72% for cellular metabolic processes, 10.56% for cellular processes, 10.33% for macromolecule metabolic processes, 7.84% for nitrogen compound metabolic processes, 7.60% for primary metabolic processes, 5.39% for RNA metabolic processes, 4.81% for heterocycle metabolic processes, and 4.79% for cellular aromatic compound metabolic processes (Fig 1). The remaining biological processes accounted for a combined total of 18.21% (Fig 1). In the molecular function category, oxidoreductase activity was the most prevalent at 25.76%, followed by transferase activity at 15.68%, hydrolase activity at 14.53%, ion binding at 7.77%, and transporter and peptidase activity each at 6.19%. Nucleic acid binding contributed 4.46%, nucleotide binding 4.16%, and general binding 3.74% (Fig 1). All other unmentioned molecular functions collectively accounted for 7.77% (Fig 1). For the cellular component category, the distributions were as follows: membrane (21%), cell part (16.89%), intracellular (10.81%), nucleus (10.81%), mitochondrion (8.11%), organelle (5.74%), intracellular organelle (5.74%), extracellular region (2.7%), and vacuole (1.71%) (Fig 1). The combined total for all other unmentioned cellular components was 16.55% (Fig 1). A similar trend in gene distribution was observed for isolate KARE80 and the *C. sorbicola* isolates (Fig 2). Figs 1 and 2 presents the representative distribution of the GO categories for both *C. plurivora* and *C. sorbicola* isolates. The relatively higher abundance of transporter and peptidase activity, each comprising approximately 6.19%, suggests an evolutionary advantage that enables the fungus to thrive in plant tissues and overcome host defenses. Peptidases are crucial hydrolytic enzymes in phytopathogenic fungi, playing significant roles in signaling, the degradation of host plant tissue, and the digestion of proteins involved in the plant’s response to pathogens [56–58]. Transporters, such as those in the major facilitator superfamily (MFS), are vital for antifungal resistance and nutrient transport, which are critical for pathogenesis in response to reactive oxygen species [22,24,59]. In related apple and pear canker pathosystems, it has been suggested that *Vasla mali* and *V. pyri* utilize membrane transporters and proteases to adapt to nutrient limitations and low pH environments in the bark [4].

### Putative virulence factors/Determinants

**Fig 3:**
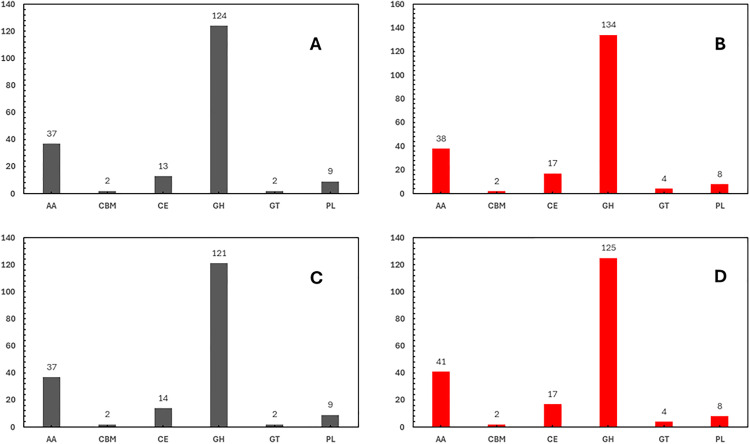
Distribution of the predicted secreted CAZymes families (A) *C. sorbicola* KARE59. **(B)** *C. plurivora* KARE79. **(C)** *C. sorbicola* KARE228. **(D)** *C. plurivora* KARE80.

CAZymes are a significant virulence factor in phytopathogenic fungi, primarily responsible for the degradation of plant cell walls during colonization [24,60,61]. The fungal secretome consists of proteins that have a signal peptide and are processed through the endoplasmic reticulum and Golgi apparatus [62]. As a result, identifying CAZymes with signal peptides is a common approach to studying potential virulence factors [18,24,63]. Therefore, this study focuses exclusively on CAZymes predicted to possess a signal peptide. For the isolates of *C. plurivora* KARE79 and KARE80, a total of 203 and 197 secreted carbohydrate-active enzymes (CAZymes) were predicted, respectively (Fig 3B, D). In the case of KARE79, the most abundant categories of these enzymes included glycoside hydrolases (GH) with 134, auxiliary activities (AA) with 38, carbohydrate esterases (CE) with 17, phospholipases (PL) with 8, glycosyltransferases (GT) with 4, and carbohydrate-binding modules (CBM) with 2 (Fig 3B). A similar distribution was noted for KARE80. The isolates of *C. sorbicola*, KARE59, and KARE228 had 185 and 187 predicted secreted CAZymes, respectively (Fig 3A, C), with their distribution across the various categories mirroring that of the *C. plurivora* isolates (Fig 3A, C).

Fungal secondary metabolites play a crucial role in mediating ecological interactions, communication, nutrient acquisition, stress protection, and chemical warfare [24,64]. Necrotrophic fungal pathogens produce various phytotoxic secondary metabolites, such as polyketides, nonribosomal peptides, terpenes, and alkaloids, to kill host cells [65]. To predict proteins related to secondary metabolite synthesis, we used the antiSMASH fungal version. The *C. plurivora* isolates KARE79 and KARE80 had a total of 71 predicted proteins associated with secondary metabolite synthesis (Table 2). The *C. sorbicola* isolates KARE59 and KARE228 had 69 and 73 predicted proteins, respectively (Table 2). The most abundant types of these proteins were polyketide synthases (PKS), followed by non-ribosomal peptide synthetases (NRPS), ribosomally synthesized and post-translationally modified peptides (RiPP), terpenes, hybrids, and others (Table 2). We also examined transporters, peptidases, and cytochromes. The total number of predicted peptidases and transporters for all *C. plurivora* and *C. sorbicola* isolates ranged from 257 to 286 (Table 2). Detailed information for each isolate can be found in Table 2. Additionally, 94–101 cytochromes were predicted for all isolates, with specific distributions presented in Table 2. Cytochromes are essential for fungal adaptations to various ecological niches, as they help to buffer harmful chemical and biological signals [19,20]. Moreover, cytochrome monooxygenases have been identified as critical virulence factors in most canker pathosystems [17–19].

**Table 2:** Predicted number of secondary metabolite synthesis encoding genes, transporters, cytochrome P450, and peptidase.

	*C. sorbicola* (KARE228)	*C. sorbicola* (KARE59)	*C. plurivora* (KARE79)	*C. plurivora* (KARE80)
	Predicted secondary metabolite protein synthesis encoding genes			
RiPP	12	13	14	11
PKS	26	25	21	22
NRPS	14	13	15	16
Terpene	10	10	8	9
Hybrid	7	10	11	9
Others	2	2	2	4
Transporters	273	274	273	286
Cytochrome P450	101	103	95	94
Peptidase	270	273	257	261

The fungal secretome comprises proteins that contain a signal peptide and are processed through the endoplasmic reticulum and Golgi apparatus. Among these proteins, effectors play a vital role in bypassing host defenses, either by concealing the presence of the pathogen or by directly killing host cells [21,62]. The total number of predicted effectors for all *C. plurivora* and *C*. *sorbicola* isolates ranged from 197 to 214. Detailed information for each isolate can be found in Table 1.

### Phylogenomic analyses

**Fig 4:**
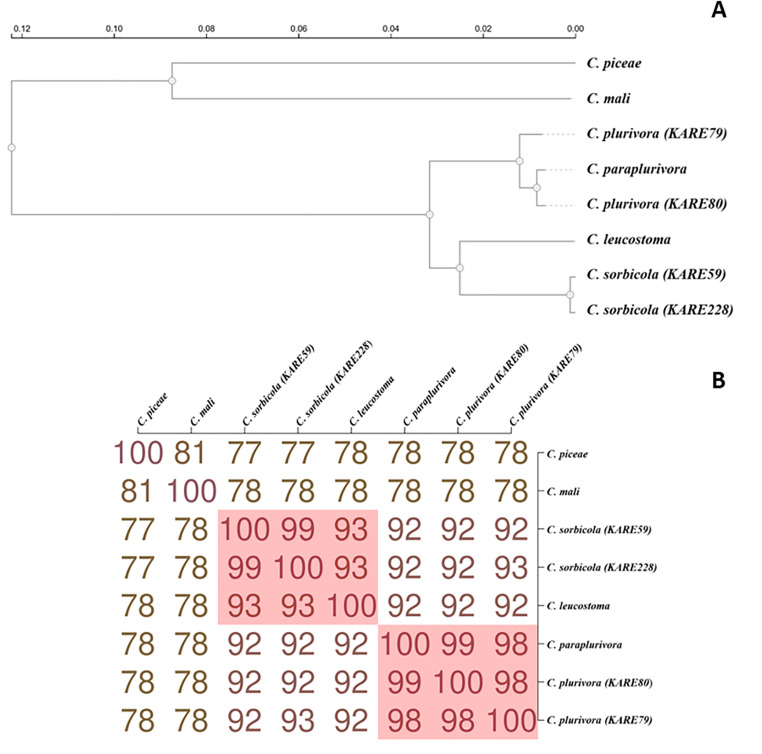
(A) A phylogenetic tree based on single-copy genes illustrates the evolutionary relationships and distances among *C. plurivora* and *C. sorbicola* isolates and the closely related species. **(B)** The genome-based distance matrix calculator calculated Average Amino Acid Identity (AAI) for *C. sorbicola* and *C. plurivora* isolates and closely related species.

**Fig 5:**
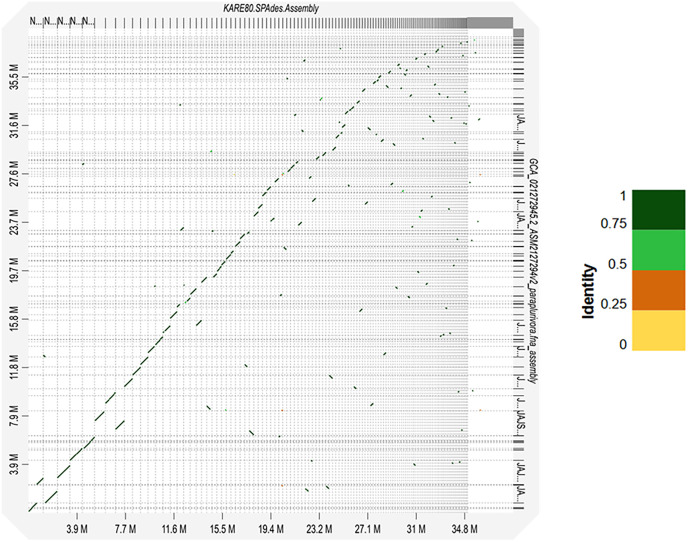
Dot plot graph showing syntenic blocks between *C. plurivora* KARE80 and *C. paraplurivora.*

**Fig 6:**
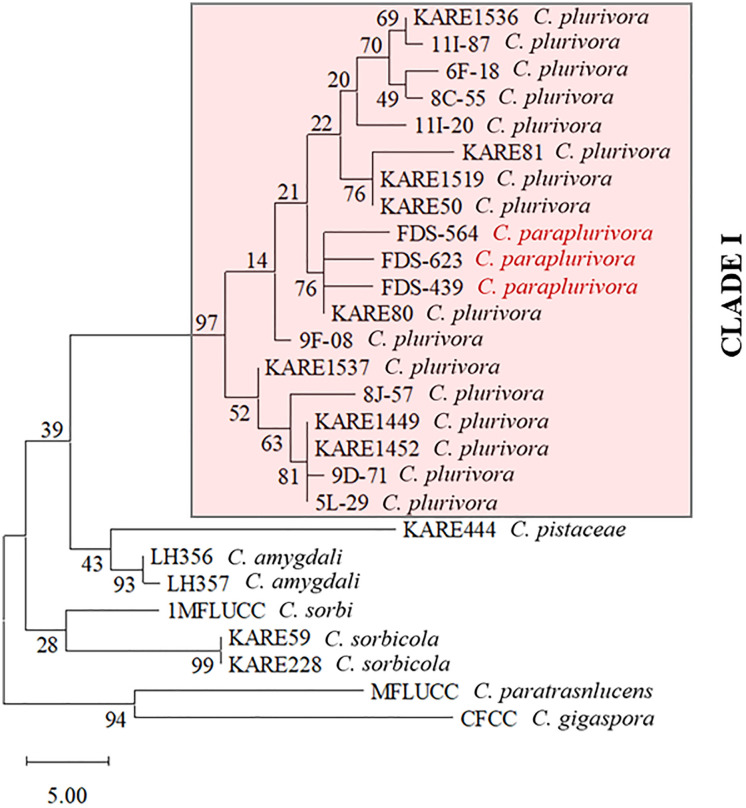
The single most parsimonious tree was generated from a maximum parsimony analysis of the combined three-gene dataset (*ITS*, *TEF1*, and *ACT1*). Bootstrap values (from 1,000 replicates) are shown next to the branches and represent the percentage of replicate trees in which the associated taxa clustered together. The analysis included 27 nucleotide sequences, and positions with less than 95% site coverage were eliminated using the partial deletion option. Isolates of *C. plurivora* representing various subclades from Lawrence et al. (2018) are designated as Clade **I.** Isolates highlighted in red within Clade I correspond *to C. paraplurivora* from Ilyukhin et al. (2023). The *C. paraplurivora* isolates formed a strongly supported clade (97%) with *C. plurivora* isolates, confirming that *C. paraplurivora* is synonymous with *C. plurivora*.

For comprehensive phylogenomic analyses, we uploaded the proteomes of the *C. plurivora* isolates KARE79 and KARE80, as well as the *C. sorbicola* isolates KARE59 and KARE228, to the OrthoVenn3 web server [46]. Additionally, we included the proteomes of *C. piceae*, *C. mali*, *C. paraplurivora*, and *C. leucostoma*. OrthoVenn3 constructs phylogenetic trees using conserved single-copy gene sequences which are independent evolutionary units among species [62]. Our analyses showed that *C. plurivora* isolates KARE79 and KARE80 formed a well-supported monophyletic clade (bootstrap value = 1) with *C. paraplurivora* (Fig 4A). Within this cohesive clade, *C. paraplurivora* formed a subclade with isolate KARE80 (bootstrap value = 1) (Fig 4A). On the other hand, the *C. sorbicola* isolates KARE59 and KARE228 formed a clade with *C. leucostoma*; however, there was minimal evidence to support this clade (Fig 4A). The *C. sorbicola* isolates themselves formed a well-supported subclade (bootstrap value = 1) (Fig 4A). These findings suggest that *C. paraplurivora* should be classified as a species within *C. plurivora*. To further verify this classification, we used a genome-based distance matrix calculator (Kostas lab | Genome matrix (gatech.edu)), which revealed that *C. paraplurivora* shares 98% and 99% Average Amino Acid Identity (AAI) with *C. plurivora* isolates KARE79 and KARE80, respectively, vividly portraying a solid species cluster (Fig 4B). Additionally, dot plots generated by D-GENIES highlighted that *C. paraplurivora* exhibited highly conserved syntenic blocks with isolates KARE79 and KARE80; in fact, an astounding 87.43% of the syntenic blocks from *C. paraplurivora* exhibited over 75% similarity with those of isolate KARE80 (Fig 5). Lawrence et al. (2018) astutely noted that *C. plurivora* contains the most genetically diverse clade among the *Cytospora* species they examined [2], reinforcing the necessity of including multiple isolates representing various subclades in phylogenetic analyses. Such an approach enhances the robustness of the findings, particularly when categorizing new species. In stark contrast, Ilyukhin et al. (2023) [31] relied on only two isolates, significantly limiting the depth of their analysis. By incorporating isolates from all subclades identified by Lawrence et al. (2018), it becomes evident that *C. paraplurivora* is synonymous with *C. plurivora* (Fig 6, S1 Fig). Compounding these challenges is the scarcity of available genome sequences for *C. plurivora*, as comprehensive whole-genome comparisons would have greatly streamlined the resolution of these intricate issues. Consequently, we propose that *C. paralurivora* be amended to *C. plurivora*, reflecting the insights gained from our analysis.

**Fig 7:**
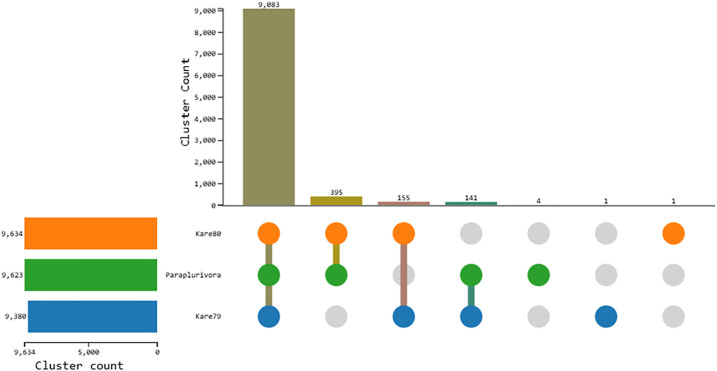
An UpSet table displaying unique and shared orthologous clusters among *C. plurivora* isolates and *C. paraplurivora.* The left horizontal bar chart shows the number of orthologous clusters per species, while the right vertical bar chart shows the number of orthologous clusters shared among the species.

Orthology is the most accurate method for describing differences and similarities in the genome composition of different species [66,67]. This is because orthologues, by definition, trace back to an ancestral gene that was present in a common ancestor of the species being compared [67]. To further assess the differences between *C. plurivora* and *C. paraplurivora*, we analyzed the shared orthologues among the *C. plurivora* isolates KARE79 and KARE80, as well as *C. paraplurivora*. The results from the OrthoVenn3 analysis indicated that the *C. plurivora* isolates and *C. paraplurivora* shared 9,083 orthologous gene clusters (Fig 7). In contrast, only four orthologues were unique to *C. paraplurivora*, which were proteins of unknown function. Additionally, *C. plurivora* isolates KARE79 and KARE80 each had one unique orthologue, also associated with proteins of unknown function (Fig 7). These results underscore the genomic similarities between *C. plurivora* and *C. paraplurivora*.

## Conclusion

In this study, we sequenced and assembled high-quality genomes of *Cytospora sorbicola* and *C. plurivora*. Currently, there are no publicly available genomes for either species, which makes our findings an important contribution as genomic resources for significant canker pathogens. We have made the assembly and raw sequences from this study publicly available. Additionally, we explored potential virulence factors, which include secreted carbohydrate-active enzymes (CAZymes), gene clusters involved in secondary metabolite biosynthesis, transporters, cytochrome genes, proteases, and putative effector proteins. The sequences of these predicted effector proteins are also publicly accessible. The primary gene predictions and annotations from our research will serve as valuable resources for expression profiling in planta, molecular information necessary for targeted knockout mutations, and studies on genetic diversity. It is important to note that experimental validation of the predicted genes is still needed, and transcriptomic experiments could enhance the utility of the resource we have presented. Furthermore, a phylogenomic analysis based on conserved single-copy gene sequences conducted in this study strongly suggests that *C. paraplurivora* should be classified under *C. plurivora*. We recommend amending the classification of *C. paraplurivora* sp. nov. (Ilyukhin et al., 2023) to *C. plurivora.* This finding has significant implications for both basic and applied research, and it can help prevent identification confusion in the future.

## Supporting information

S1 Fig Maximum Likelihood phylogeny based on a combined dataset of three genes (ITS, TEF1, and ACT1). The percentages below the branches indicate the proportion of replicate trees (1,000 replicates) in which the associated taxa clustered together. For the heuristic search, the initial tree was chosen based on the higher log-likelihood between a Neighbor-Joining (NJ) tree and a Maximum Parsimony (MP) tree. The NJ tree was constructed using pairwise distances calculated with the Tamura-Nei model. The MP tree was the shortest among 10 searches, each starting from a randomly generated tree. Isolates of *C. plurivora* representing different subclades from Lawrence et al. (2018) are shown as Clade I. Isolates highlighted in red within Clade I correspond to *C. paraplurivora* from Ilyukhin et al. (2023). *The C. paraplurivora* isolates formed a strongly supported clade (97%) with *C. plurivora* isolates, indicating that *C. paraplurivora* is synonymous with *C. plurivora*.
(TIF)
