# Supplementary figures and images for: Genome sequencing and analysis of isolates of Cytospora sorbicola and Cytospora plurivora associated with almond and peach canker
Source: PLoS One. 2025 Oct 17;20(10):e0334178. doi: 10.1371/journal.pone.0334178 (PMC12533866; doi:10.1371/journal.pone.0334178)

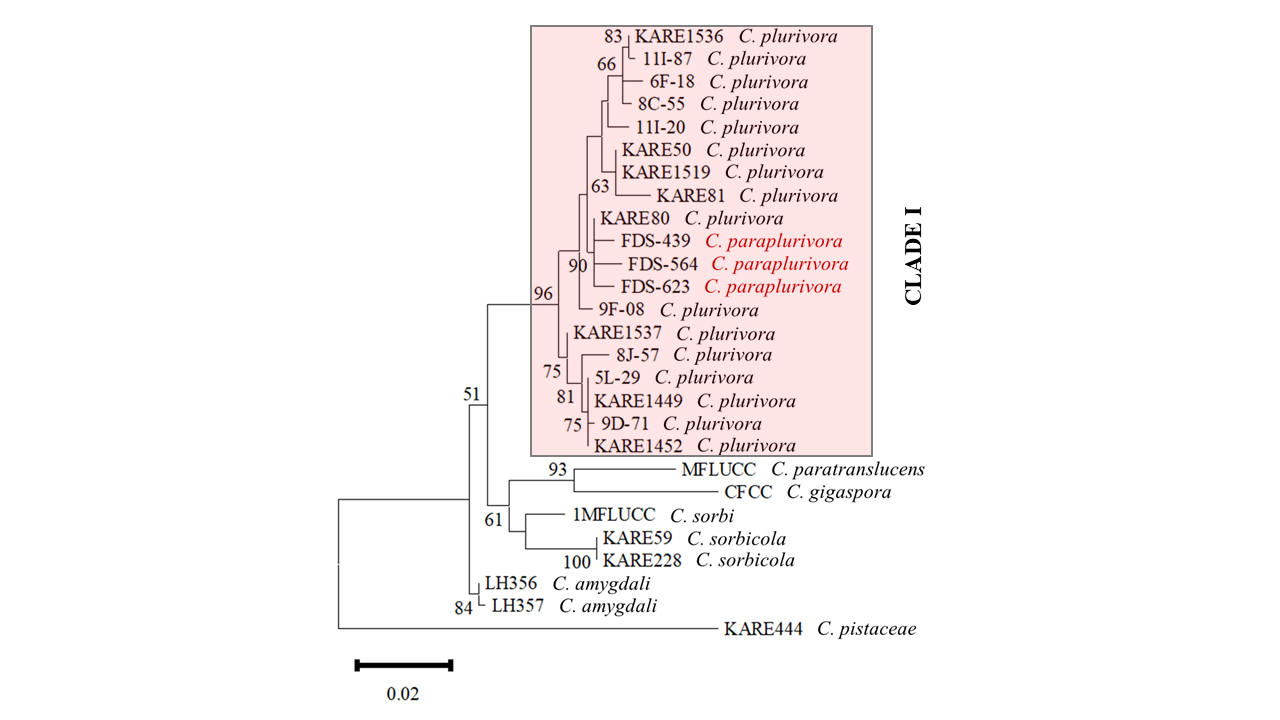

Supplement: S1 Fig — The percentages below the branches indicate the proportion of replicate trees (1,000 replicates) in which the associated taxa clustered together. For the heuristic search, the initial tree was chosen based on the higher log-likelihood between a Neighbor-Joining (NJ) tree and a Maximum Parsimony (MP) tree. The NJ tree was constructed using pairwise distances calculated with the Tamura-Nei model. The MP tree was the shortest among 10 searches, each starting from a randomly generated tree. Isolates of C. plurivora representing different subclades from Lawrence et al. (2018) are shown as Clade I. Isolates highlighted in red within Clade I correspond to C. paraplurivora from Ilyukhin et al. (2023). The C. paraplurivora isolates formed a strongly supported clade (97%) with C. plurivora isolates, indicating that C. paraplurivora is synonymous with C. plurivora. (TIF) [file pone.0334178.s001.tif]
